# Supplementary figures and images for: Human monoclonal antibodies against Staphylococcus aureus surface antigens recognize in vitro and in vivo biofilm
Source: eLife. 2022 Jan 6;11:e67301. doi: 10.7554/eLife.67301 (PMC8751199; doi:10.7554/eLife.67301)

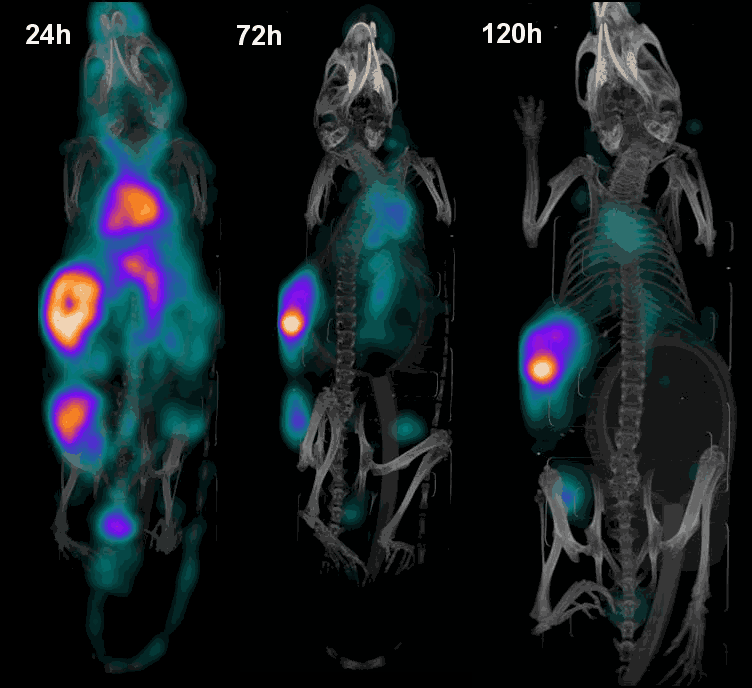

Supplement: Supplementary file 1 [file elife-67301-fig7-video1.gif]

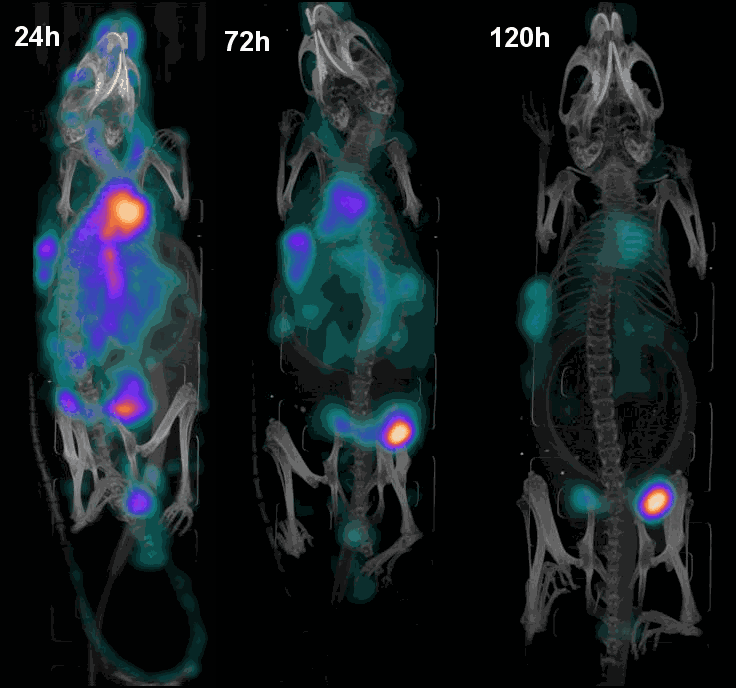

Supplement: Supplementary file 2 [file elife-67301-fig7-video2.gif]
