# Supplementary material for: Human monoclonal antibodies against Staphylococcus aureus surface antigens recognize in vitro and in vivo biofilm
Source: eLife. 2022 Jan 6;11:e67301. doi: 10.7554/eLife.67301 (PMC8751199; doi:10.7554/eLife.67301)
Supplement: Supplementary file 1. [file elife-67301-supp1.docx]

Supplementary File 1. Protein sequences used for human monoclonal antibody production.

| **Clone, *target*** | **Sequence** | **Reference** |
| --- | --- | --- |
| **VH variable heavy chain** | | |
| G2a2,  *Anti-DNP* | DVRLQESGPGLVKPSQSLSLTCSVTGYSITNSYYWNWIRQFPGNKLEWMVYIGYDGSNNYNPSLKNRISITRDTSKNQFFLKLNSVTTEDTATYYCARATYYGNYRGFAYWGQGTLVTVSA | Gonzalez 2003 (41) |
| B12,  *Anti-gp120* | QVQLVQSGAEVKKPGASVKVSCQASGYRFSNFVIHWVRQAPGQRFEWMGWINPYNGNKEFSAKFQDRVTFTADTSANTAYMELRSLRSADTAVYYCARVGPYSWDDSPQDNYYMDVWGKGTTVIVSS | Barbas 1993 (42)  Saphire 2001 (86) |
| 4461,  *Anti-WTA(α)* | QVQLVQSGAEVRKPGASVKVSCKASGYSFTDYYMHWVRQAPGQGLEWMGWINPKSGGTNYAQRFQGRVTMTGDTSISAAYMDLASLTSDDTAVYYCVKDCGSGGLRDFWGQGTTVTVSS | WO/2014/193722 A1 |
| 4497,  *Anti-WTA(β)* | EVQLVESGGGLVQPGGSLRLSCSASGFSFNSFWMHWVRQVPGKGLVWISFTNNEGTTTAYADSVRGRFIISRDNAKNTLYLEMNNLRGEDTAVYYCARGDGGLDDWGQGTLVTVSS. | WO/2014/193722 A1  Lehar 2015 (38)  Fong 2018 (39) |
| CR5132 | EVLESGGGLVQPGGSLRLSCSDSGFSFNNYWMTWVRQAPGKGLEWVANINRDGSDKYHVDSVEGRFTISRDNSKNSLYLQMNNLRADDAA VYFCARGGRTTSWYWRNWGQGTLVTVSS | US 2012/0141493 A1 |
| F598,  *Anti-PNAG* | QVQLQESGPGLVKPSETLSLTCTVSGGSISGYYWSWIRQPPGKGLEWIGYIHYSRSTNSNPALKSRVTISSDTSKNQLSLRLSSVTAADTAVYYCARDTYYYDSGDYEDAFDIWGQGTMVTVSS | US/2006/0115486 A1 Seq25  Kelly-Quintos 2006 (73)  Soliman 2018 (21) |
| rF1,  *Anti-GlcNac pan-SDR* | EVQLVESGGGLVQPGGSLRLSCAASGFTLSRFAMSWVRQAPGRGLEWVASINSGNNPYYARSVQYRFTVSRDVSQNTVSLQMNNLRAEDSATYFCAKDHPSSGWPTFDSWGPGTLVTVSS | WO/2016/090040 Seq13  Hazenbos 2013 (40) |
| T1-2,  *Anti-ClfA* | QVQLKESGPGLVAPSQSLSITCAISGFSLSRYSVHWVRQPPGKGLEWLGMIWGGGNTDYNSALKSRLSISKDNSKSQVFLKMNSLQTDDTAMYYCARKGEFYYGYDGFVYWGQGTLVTVSA | WO 02072600 A2 |
| A120,  *Anti-LTA* | EVMLVESGGGLVQPKGSLKLSCAASGFTFNTYAMNWVRQAPGKGLEWVARIRSKSNNYATYYADSVKDRFTISRDDSQSMLYLQMNNLKTEDTAMYYCVRRGGKETDYAM DYWGQGTSVT VSS | WO 03/059259 |
| 10919  *Anti-SpA* | EVQLVQSGAEVKKPGASVKVSCKASGYTFTSYYMHWVRQAPGQGLEWMGIINPRVGSTSYAQKFQGRVTMTRDTSTSTVYMELSSLRSEDTAVYYCARGRPLSGTGGHHYFDYWGQGTLVTVSS | US2018/0105584 |
| **VL variable light chain** | | |
| G2a2,  *Anti-DNP* | DIRMTQTTSSLSASLGDRVTISCRASQDISNYLNWYQQKPDGTVKLLIYYTSRLHSGVPSRFSGSGSGTDYSLTISNLEQEDIATYFCQQGNTLPWTFGGGTKLEIK | Gonzalez 2003 (41) |
| B12,  *Anti-gp120* | EIVLTQSPGTLSLSPGERATFSCRSSHSIRSRRVAWYQHKPGQAPRLVIHGVSNRASGISDRFSGSGSGTDFTLTITRVEPEDFALYYCQVYGASSYTFGQGTKLERK | Barbas 1993 (42)  Saphire 2001 (86) |
| 4461,  *Anti-WTA(α)* | DIQMTQSPDSLAVSLGERATINCKSSQSVLSRANNNYYVAWYQHKPGQPPKLLIYWASTREFGVPDRFSGSGSGTDFTLTINSLQAEDVAVYYCQQYYTSRRTFGQGTKVEIK | WO/2014/193722 A1 |
| 4497,  *Anti-WTA(β)* | DIQLTQSPDSLAVSLGERATINCKSSQSIFRTSRNKNLLNWYQQRPGQPPRLLIHWASTRKSGVPDRFSGSGFGTDFTLTITSLQAEDVAIYYCQQYFSPPYTFGQGTKLEIK | WO/2014/193722 A1  Lehar 2015 (38)  Fong 2018 (39) |
| CR5132 | STDIQMTQSPSTLSASVGDRVTITCRASQSISSWLAWYQQKPGKAPKLLIYKASSLESGVPSRFSGSGSGTEFTLTISSLQPDDFATYYC QQYNSYPLTFGGGTKLEIK | US 2012/0141493 A1 |
| F598,  *Anti-PNAG* | QLVLTQSPSASASLGASVKLTCTLSSGHSNYAIAWHQQQPGKGPRYLMKVNRDGSHIRGDGIPDRFSGSTSGAERYLTISSLQSEDEADYYCQTWGAGIRVFGGGTKLTVLG | US/2006/0115486 A1 Seq 26  Kelly-Quintos 2006 (73)  Soliman 2018 (21) |
| rF1,  *Anti-GlcNac pan-SDR* | DIQLTQSPSALPASVGDRVSITCRASENVGDWLAWYRQKPGKAPNLLIYKTSILESGVPSRFSGSGSGTEFTLTISSLQPDDFATYYCQHYMRFPYTFGQGTKVEIK | WO/2016/090040_Seq14  Hazenbos 2013 (40) |
| T1-2,  *Anti-ClfA* | NIMMTQSPSSLAVSAGEKVTMSCKSSQSVLYSSNQKNYLAWYQQKPGQSPKLLIYWASTRESGVPDRFTGSGSGTDFTLTISSVQAEDLAVYYCHQYLSSYTFGGGTKLEIK | WO 02072600 A2 |
| A120,  *Anti-LTA* | DIVLSQSPAILSASPGEKVTMTCRASSSVSYMHWYQQKPGSSPKPWIYATSNLASGVPARFSGSGSGTSYSLTISRVEAEDAATYYCQQWSSNPPTFGGGTKLEIK | WO 03/059259 |
| 10919  *Anti-SpA* | EIVLTQSPATLSVSPGERATLSCQASQDISNYLNWYQQKPGQAPRLLIYDASNLETGIPARFSGSGSGTEFTLTISSLQSEDFAVYYCQQVYALPPWTFGGGTKVEIK | US2018/0105584 |
| **HC constant regions** | | |
| IgG1 | ASTKGPSVFPLAPSSKSTSGGTAALGCLVKDYFPEPVTVSWNSGALTSGVHTFPAVLQSSGLYSLSSVVTVPSSSLGTQTYICNVNHKPSNTKVDKKVEPKSCDKTHTCPPCPAPELLGGPSVFLFPPKPKDTLMISRTPEVTCVVVDVSHEDPEVKFNWYVDGVEVHNAKTKPREEQYNSTYRVVSVLTVLHQDWLNGKEYKCKVSNKALPAPIEKTISKAKGQPREPQVYTLPPSREEMTKNQVSLTCLVKGFYPSDIAVEWESNGQPENNYKTTPPVLDSDGSFFLYSKLTVDKSRWQQGNVFSCSVMHEALHNHYTQKSLSLSPGK | Kabat 1991 (87) |
| IgG3 | ASTKGPSVFPLAPCSRSTSGGTAALGCLVKDYFPEPVTVSWNSGALTSGVHTFPAVLQSSGLYSLSSVVTVPSSSLGTQTYTCNVNHKPSNTKVDKRVELKTPLGDTTHTCPRCPEPKSCDTPPPCPRCPEPKSCDTPPPCPRCPEPKSCDTPPPCPRCPAPELLGGPSVFLFPPKPKDTLMISRTPEVTCVVVDVSHEDPEVQFKWYVDGVEVHNAKTKPREEQYNSTFRVVSVLTVLHQDWLNGKEYKCKVSNKALPAPIEKTISKTKGQPREPQVYTLPPSREEMTKNQVSLTCLVKGFYPSDIAVEWESSGQPENNYNTTPPMLDSDGSFFLYSKLTVDKSRWQQGNIFSCSVMHEALHNRFTQKSLSLSPGK | Derived from pFuse vector (Invivogen) |
| **LC constant regions** | | |
| Kappa class | RTVAAPSVFIFPPSDEQLKSGTASVVCLLNNFYPREAKVQWKVDNALQSGNSQESVTEQDSKDSTYSLSSTLTLSKADYEKHKVYACEVTHQGLSSPVTKSFNRGEC | Kabat 1991 (87) |
