# Supplementary material for: Human monoclonal antibodies against Staphylococcus aureus surface antigens recognize in vitro and in vivo biofilm
Source: eLife. 2022 Jan 6;11:e67301. doi: 10.7554/eLife.67301 (PMC8751199; doi:10.7554/eLife.67301)
Supplement: Supplementary file 2. [file elife-67301-supp2.docx]

**Supplementary File 2**

| **Fig** |  | **Description** | **Comparison** | **Test** | **Summary** | **P-value** |
| --- | --- | --- | --- | --- | --- | --- |
| 1 | B | Wood46 biofilm F598 | ctrl vs f598 | ratio paired t-test | * | 0,0343 |
| 1 | C | LAC KO biofilm F598 | ctrl vs f598 | ratio paired t-test | ns | 0,4025 |
| 5 | A | Wood biofilm binding AUC | Ctrl vs. F598 (PNAG) | ordinary one-way ANOVA, dunnett | *** | 0,0001 |
| 5 | A | Wood biofilm binding AUC | Ctrl vs. 4461 (α) | ordinary one-way ANOVA, dunnett | ns | 0,7568 |
| 5 | A | Wood biofilm binding AUC | Ctrl vs. 4497 (β) | ordinary one-way ANOVA, dunnett | **** | <0,0001 |
| 5 | A | Wood biofilm binding AUC | Ctrl vs. rF1 (SDR) | ordinary one-way ANOVA, dunnett | ** | 0,0013 |
| 5 | A | Wood biofilm binding AUC | Ctrl vs. T1-2 (ClfA) | ordinary one-way ANOVA, dunnett | ns | 0,6004 |
| 5 | A | Wood biofilm binding AUC | Ctrl vs. CR5132 (β) | ordinary one-way ANOVA, dunnett | ** | 0,0012 |
| 5 | B | LAC biofilm binding auc | Ctrl vs. F598 (PNAG) | ordinary one-way ANOVA, dunnett | ns | >0,9999 |
| 5 | B | LAC biofilm binding auc | Ctrl vs. 4461 (α) | ordinary one-way ANOVA, dunnett | ** | 0,002 |
| 5 | B | LAC biofilm binding auc | Ctrl vs. 4497 (β) | ordinary one-way ANOVA, dunnett | * | 0,0379 |
| 5 | B | LAC biofilm binding auc | Ctrl vs. rF1 (SDR) | ordinary one-way ANOVA, dunnett | ** | 0,008 |
| 5 | B | LAC biofilm binding auc | Ctrl vs. T1-2 (ClfA) | ordinary one-way ANOVA, dunnett | * | 0,0224 |
| 5 | B | LAC biofilm binding auc | Ctrl vs. CR5132 (β) | ordinary one-way ANOVA, dunnett | *** | 0,0001 |
| 6 | A | Clinical isolates F598 binding | LAC KO vs. D1524 | ordinary one-way ANOVA, dunnett | ns | 0,9982 |
| 6 | A | Clinical isolates F598 binding | LAC KO vs. D1897 | ordinary one-way ANOVA, dunnett | ns | 0,9996 |
| 6 | A | Clinical isolates F598 binding | LAC KO vs. D2085 | ordinary one-way ANOVA, dunnett | ns | 0,9997 |
| 6 | A | Clinical isolates F598 binding | LAC KO vs. D2457 | ordinary one-way ANOVA, dunnett | ns | 0,999 |
| 6 | A | Clinical isolates F598 binding | LAC KO vs. D463 | ordinary one-way ANOVA, dunnett | ** | 0,0034 |
| 6 | A | Clinical isolates F598 binding | LAC KO vs. D558 | ordinary one-way ANOVA, dunnett | ns | 0,9991 |
| 6 | A | Clinical isolates F598 binding | LAC KO vs. E201 | ordinary one-way ANOVA, dunnett | ns | 0,9996 |
| 6 | A | Clinical isolates F598 binding | LAC KO vs. E2187 | ordinary one-way ANOVA, dunnett | ns | 0,9997 |
| 6 | A | Clinical isolates F598 binding | LAC KO vs. E2259 | ordinary one-way ANOVA, dunnett | ns | 0,9997 |
| 6 | A | Clinical isolates F598 binding | LAC KO vs. E2332 | ordinary one-way ANOVA, dunnett | ns | 0,9997 |
| 6 | A | Clinical isolates F598 binding | LAC KO vs. F955 | ordinary one-way ANOVA, dunnett | ns | 0,9998 |
| 6 | A | Clinical isolates F598 binding | LAC KO vs. K4308 | ordinary one-way ANOVA, dunnett | ns | 0,9997 |
| 6 | A | Clinical isolates F598 binding | LAC KO vs. G348 | ordinary one-way ANOVA, dunnett | * | 0,0139 |
| 6 | A | Clinical isolates F598 binding | LAC KO vs. H1308 | ordinary one-way ANOVA, dunnett | ns | 0,998 |
| 6 | A | Clinical isolates F598 binding | LAC KO vs. H2884 | ordinary one-way ANOVA, dunnett | ns | 0,2159 |
| 6 | A | Clinical isolates F598 binding | LAC KO vs. J182 | ordinary one-way ANOVA, dunnett | ns | 0,9997 |
| 6 | A | Clinical isolates F598 binding | LAC KO vs. J4723 | ordinary one-way ANOVA, dunnett | **** | <0,0001 |
| 6 | A | Clinical isolates F598 binding | LAC KO vs. L1660 | ordinary one-way ANOVA, dunnett | ** | 0,0022 |
| 6 | A | Clinical isolates F598 binding | LAC KO vs. M0229 | ordinary one-way ANOVA, dunnett | ns | 0,9996 |
| 6 | A | Clinical isolates F598 binding | LAC KO vs. M1331 | ordinary one-way ANOVA, dunnett | ns | 0,9996 |
| 6 | A | Clinical isolates F598 binding | LAC KO vs. M2198 | ordinary one-way ANOVA, dunnett | ns | 0,9999 |
| 6 | A | Clinical isolates F598 binding | LAC KO vs. M4601 | ordinary one-way ANOVA, dunnett | * | 0,0223 |
| 6 | A | Clinical isolates F598 binding | LAC KO vs. N1766 | ordinary one-way ANOVA, dunnett | ns | 0,9998 |
| 6 | A | Clinical isolates F598 binding | LAC KO vs. N1937 | ordinary one-way ANOVA, dunnett | ns | >0,9999 |
| 6 | A | Clinical isolates F598 binding | LAC KO vs. P0759 | ordinary one-way ANOVA, dunnett | ns | 0,9997 |
| 6 | A | Clinical isolates F598 binding | LAC KO vs. F366 | ordinary one-way ANOVA, dunnett | ns | 0,271 |
| 6 | A | Clinical isolates F598 binding | LAC KO vs. F492 | ordinary one-way ANOVA, dunnett | ns | >0,9999 |
| 6 | A | Clinical isolates F598 binding | LAC KO vs. F982 | ordinary one-way ANOVA, dunnett | *** | 0,0004 |
| 6 | A | Clinical isolates F598 binding | LAC KO vs. G1560 | ordinary one-way ANOVA, dunnett | ns | 0,9997 |
| 6 | A | Clinical isolates F598 binding | LAC KO vs. K4705 | ordinary one-way ANOVA, dunnett | ** | 0,0081 |
| 6 | A | Clinical isolates F598 binding | LAC KO vs. K5171 | ordinary one-way ANOVA, dunnett | **** | <0,0001 |
| 6 | A | Clinical isolates F598 binding | LAC KO vs. K5745 | ordinary one-way ANOVA, dunnett | ns | 0,9998 |
| 6 | A | Clinical isolates F598 binding | LAC KO vs. L0977 | ordinary one-way ANOVA, dunnett | *** | 0,0001 |
| 6 | A | Clinical isolates F598 binding | LAC KO vs. L1641 | ordinary one-way ANOVA, dunnett | ns | 0,9997 |
| 6 | A | Clinical isolates F598 binding | LAC KO vs. L2607 | ordinary one-way ANOVA, dunnett | ns | >0,9999 |
| 6 | A | Clinical isolates F598 binding | LAC KO vs. L2717 | ordinary one-way ANOVA, dunnett | ns | 0,9999 |
| 6 | A | Clinical isolates F598 binding | LAC KO vs. L4910 | ordinary one-way ANOVA, dunnett | ns | 0,9998 |
| 6 | A | Clinical isolates F598 binding | LAC KO vs. N0307 | ordinary one-way ANOVA, dunnett | **** | <0,0001 |
| 6 | A | Clinical isolates F598 binding | LAC KO vs. N0395 | ordinary one-way ANOVA, dunnett | ns | 0,0958 |
| 6 | A | Clinical isolates F598 binding | LAC KO vs. N3263 | ordinary one-way ANOVA, dunnett | ns | 0,9999 |
| 6 | A | Clinical isolates F598 binding | LAC KO vs. N4496 | ordinary one-way ANOVA, dunnett | **** | <0,0001 |
| 6 | A | Clinical isolates F598 binding | LAC KO vs. N4823 | ordinary one-way ANOVA, dunnett | ns | 0,5845 |
| 6 | A | Clinical isolates F598 binding | LAC KO vs. P0320 | ordinary one-way ANOVA, dunnett | ns | >0,9999 |
| 6 | A | Clinical isolates F598 binding | LAC KO vs. P0958 | ordinary one-way ANOVA, dunnett | ** | 0,0029 |
| 6 | A | Clinical isolates F598 binding | LAC KO vs. P1090 | ordinary one-way ANOVA, dunnett | ns | 0,999 |
| 6 | A | Clinical isolates F598 binding | LAC KO vs. Wood46 | ordinary one-way ANOVA, dunnett | **** | <0,0001 |
| 7 | B | in vivo % 24h | Pal sterile vs. Pal colonized | two-tailed paired t-test | ns | 0,1549 |
| 7 | B | in vivo % 24h | 4497 sterile vs. 4497 colonized | two-tailed paired t-test | ** | 0,0074 |
| 7 | B | in vivo % 72h | Pal sterile vs. Pal colonized | two-tailed paired t-test | ns | 0,1997 |
| 7 | B | in vivo % 72h | 4497 sterile vs. 4497 colonized | two-tailed paired t-test | ** | 0,0063 |
| 7 | B | in vivo % 120h | Pal sterile vs. Pal colonized | two-tailed paired t-test | ns | 0,1917 |
| 7 | B | in vivo % 120h | 4497 sterile vs. 4497 colonized | two-tailed paired t-test | ** | 0,0077 |
| S1 | B | DspB DNase Wood biofilm | Buffer vs. DspB | ordinary one-way ANOVA, dunnett | ** | 0,0045 |
| S1 | B | DspB DNase Wood biofilm | Buffer vs. DNase | ordinary one-way ANOVA, dunnett | ns | 0,7769 |
| S1 | A | DspB DNase LAC biofilm | Buffer vs. DspB | ordinary one-way ANOVA, dunnett | ns | 0,4974 |
| S1 | A | DspB DNase LAC biofilm | Buffer vs. DNase | ordinary one-way ANOVA, dunnett | **** | <0,0001 |
| S11 | A | LAC WT planktonic binding AUC | Ctrl vs. 4461 (α) | ordinary one-way ANOVA, dunnett | ** | 0,0014 |
| S11 | A | LAC WT planktonic binding AUC | Ctrl vs. 4497 (β) | ordinary one-way ANOVA, dunnett | ** | 0,0015 |
| S11 | A | LAC WT planktonic binding AUC | Ctrl vs. rF1 (SDR) | ordinary one-way ANOVA, dunnett | **** | <0,0001 |
| S11 | A | LAC WT planktonic binding AUC | Ctrl vs. T1-2 (ClfA) | ordinary one-way ANOVA, dunnett | ns | 0,4661 |
| S11 | A | LAC WT planktonic binding AUC | Ctrl vs. CR5132 (β) | ordinary one-way ANOVA, dunnett | ** | 0,0025 |
| S11 | B | LAC WT biofilm binding AUC | Ctrl vs. F598 (PNAG) | ordinary one-way ANOVA, dunnett | ns | >0,9999 |
| S11 | B | LAC WT biofilm binding AUC | Ctrl vs. 4461 (α) | ordinary one-way ANOVA, dunnett | **** | <0,0001 |
| S11 | B | LAC WT biofilm binding AUC | Ctrl vs. 4497 (β) | ordinary one-way ANOVA, dunnett | **** | <0,0001 |
| S11 | B | LAC WT biofilm binding AUC | Ctrl vs. rF1 (SDR) | ordinary one-way ANOVA, dunnett | **** | <0,0001 |
| S11 | B | LAC WT biofilm binding AUC | Ctrl vs. T1-2 (ClfA) | ordinary one-way ANOVA, dunnett | * | 0,011 |
| S11 | B | LAC WT biofilm binding AUC | Ctrl vs. CR5132 (β) | ordinary one-way ANOVA, dunnett | **** | <0,0001 |
| S8 | A | wood planktonic binding auc | Ctrl vs. F598 (PNAG) | ordinary one-way ANOVA, dunnett | ns | 0,9981 |
| S8 | A | wood planktonic binding auc | Ctrl vs. 4461 (α) | ordinary one-way ANOVA, dunnett | ns | >0,9999 |
| S8 | A | wood planktonic binding auc | Ctrl vs. 4497 (β) | ordinary one-way ANOVA, dunnett | **** | <0,0001 |
| S8 | A | wood planktonic binding auc | Ctrl vs. rF1 (SDR) | ordinary one-way ANOVA, dunnett | **** | <0,0001 |
| S8 | A | wood planktonic binding auc | Ctrl vs. T1-2 (ClfA) | ordinary one-way ANOVA, dunnett | ns | 0,9343 |
| S8 | A | wood planktonic binding auc | Ctrl vs. CR5132 (β) | ordinary one-way ANOVA, dunnett | ns | 0,1131 |
| S8 | B | LAC planktonic binding auc | Ctrl vs. F598 (PNAG) | ordinary one-way ANOVA, dunnett | ns | >0,9999 |
| S8 | B | LAC planktonic binding auc | Ctrl vs. 4461 (α) | ordinary one-way ANOVA, dunnett | **** | <0,0001 |
| S8 | B | LAC planktonic binding auc | Ctrl vs. 4497 (β) | ordinary one-way ANOVA, dunnett | ns | 0,653 |
| S8 | B | LAC planktonic binding auc | Ctrl vs. rF1 (SDR) | ordinary one-way ANOVA, dunnett | **** | <0,0001 |
| S8 | B | LAC planktonic binding auc | Ctrl vs. T1-2 (ClfA) | ordinary one-way ANOVA, dunnett | ns | 0,2564 |
| S8 | B | LAC planktonic binding auc | Ctrl vs. CR5132 (β) | ordinary one-way ANOVA, dunnett | * | 0,0424 |
