# Supplementary material for: Human monoclonal antibodies against Staphylococcus aureus surface antigens recognize in vitro and in vivo biofilm
Source: eLife. 2022 Jan 6;11:e67301. doi: 10.7554/eLife.67301 (PMC8751199; doi:10.7554/eLife.67301)
Supplement: Transparent reporting form [file elife-67301-transrepform1.docx]

***eLife’s* transparent reporting form**

We encourage authors to provide detailed information *within their submission* to facilitate the interpretation and replication of experiments. Authors can upload supporting documentation to indicate the use of appropriate reporting guidelines for health-related research (see [EQUATOR Network](http://www.equator-network.org/%20)), life science research (see the [BioSharing Information Resource](https://biosharing.org/)), or the [ARRIVE guidelines](http://www.plosbiology.org/article/info:doi/10.1371/journal.pbio.1000412) for reporting work involving animal research. Where applicable, authors should refer to any relevant reporting standards documents in this form.

If you have any questions, please consult our Journal Policies and/or contact us: [editorial@elifesciences.org](mailto:editorial@elifesciences.org).

**Sample-size estimation**

- You should state whether an appropriate sample size was computed when the study was being designed
- You should state the statistical method of sample size computation and any required assumptions
- If no explicit power analysis was used, you should describe how you decided what sample (replicate) size (number) to use

Please outline where this information can be found within the submission (e.g., sections or figure legends), or explain why this information doesn’t apply to your submission:

The information on the sample sizes of all experiments can be found in the figure legends and the material and methods section.

For animal studies, the group size was determined by a power analysis that was based on experimental variation obtained in a pilot study (using G*Power 3.1 software) aiming for a power of 0.95. A minimum of 4 mice per group was calculated based on the expected difference between 4497-IgG1 localization to sterile implants versus pre-colonized implants.

*In vitro* experiments were replicated at least three times via independent experiments (unless stated otherwise). The sample size of N=3 has traditionally been employed in *in vitro* bacterial studies and therefore we have also chosen this sample size.

**Replicates**

- You should report how often each experiment was performed
- You should include a definition of biological versus technical replication
- The data obtained should be provided and sufficient information should be provided to indicate the number of independent biological and/or technical replicates
- If you encountered any outliers, you should describe how these were handled
- Criteria for exclusion/inclusion of data should be clearly stated
- High-throughput sequence data should be uploaded before submission, with a private link for reviewers provided (these are available from both GEO and ArrayExpress)

Please outline where this information can be found within the submission (e.g., sections or figure legends), or explain why this information doesn’t apply to your submission:

The number of technical replicates (experiment performed in duplicate/triplicate) and biological replicates (number of independent experiments) is indicated in each figure legend.

**Statistical reporting**

- Statistical analysis methods should be described and justified
- Raw data should be presented in figures whenever informative to do so (typically when N per group is less than 10)
- For each experiment, you should identify the statistical tests used, exact values of N, definitions of center, methods of multiple test correction, and dispersion and precision measures (e.g., mean, median, SD, SEM, confidence intervals; and, for the major substantive results, a measure of effect size (e.g., Pearson's r, Cohen's d)
- Report exact p-values wherever possible alongside the summary statistics and 95% confidence intervals. These should be reported for all key questions and not only when the p-value is less than 0.05.

Please outline where this information can be found within the submission (e.g., sections or figure legends), or explain why this information doesn’t apply to your submission:

Information regarding statistical analysis is included into the material and methods section and in the figure legends and in Supplementary File 2.

(For large datasets, or papers with a very large number of statistical tests, you may upload a single table file with tests, Ns, etc., with reference to sections in the manuscript.)

**Group allocation**

- Indicate how samples were allocated into experimental groups (in the case of clinical studies, please specify allocation to treatment method); if randomization was used, please also state if restricted randomization was applied
- Indicate if masking was used during group allocation, data collection and/or data analysis

Please outline where this information can be found within the submission (e.g., sections or figure legends), or explain why this information doesn’t apply to your submission:

The analyzing investigator was blinded for the injection of [111In]-4497-IgG1 or [111In]-Palivizumab and the implantation of sterile and pre-colonized catheters in the left or the right flank was randomized. This information can be found in the methods section.

**Additional data files (“source data”)**

- We encourage you to upload relevant additional data files, such as numerical data that are represented as a graph in a figure, or as a summary table
- Where provided, these should be in the most useful format, and they can be uploaded as “Source data” files linked to a main figure or table
- Include model definition files including the full list of parameters used
- Include code used for data analysis (e.g., R, MatLab)
- Avoid stating that data files are “available upon request”

Please indicate the figures or tables for which source data files have been provided:

Source data for all figures, Z stacks and mouse scans have been uploaded to Dryad:

https://datadryad.org/stash/share/W5JokHBjsFcKy6Z4BVLhx4JuDdfzJ1KrxHSGkphde84
